# Convolutional Neural Network for Skin Lesion Classification: Understanding the Fundamentals Through Hands-On Learning

**DOI:** 10.3389/fmed.2021.644327

**Published:** 2021-03-04

**Authors:** Marta Cullell-Dalmau, Sergio Noé, Marta Otero-Viñas, Ivan Meić, Carlo Manzo

**Affiliations:** ^1^The QuBI Lab, Facultat de Ciències i Tecnologia, Universitat de Vic – Universitat Central de Catalunya, Vic, Spain; ^2^Tissue Repair and Regeneration Laboratory, Facultat de Ciències i Tecnologia, Universitat de Vic – Universitat Central de Catalunya, Vic, Spain; ^3^University of Zagreb, Zagreb, Croatia

**Keywords:** convolutional neural networks, skin lesion analysis, classification, melanoma, deep learning

## Abstract

Deep learning architectures for the classification of images have shown outstanding results in a variety of disciplines, including dermatology. The expectations generated by deep learning for, e.g., image-based diagnosis have created the need for non-experts to become familiar with the working principles of these algorithms. In our opinion, getting hands-on experience with these tools through a simplified but accurate model can facilitate their understanding in an intuitive way. The visualization of the results of the operations performed by deep learning algorithms on dermatological images can help students to grasp concepts like convolution, even without an advanced mathematical background. In addition, the possibility to tune hyperparameters and even to tweak computer code further empower the reach of an intuitive comprehension of these processes, without requiring advanced computational and theoretical skills. This is nowadays possible thanks to recent advances that have helped to lower technical and technological barriers associated with the use of these tools, making them accessible to a broader community. Therefore, we propose a hands-on pedagogical activity that dissects the procedures to train a convolutional neural network on a dataset containing images of skin lesions associated with different skin cancer categories. The activity is available open-source and its execution does not require the installation of software. We further provide a step-by-step description of the algorithm and of its functions, following the development of the building blocks of the computer code, guiding the reader through the execution of a realistic example, including the visualization and the evaluation of the results.

## Introduction: Background and Rationale for The Educational Activity Innovation

Over the last two decades, convolutional neural networks (CNNs) (1) have become established as an invaluable tool for biomedical image classification and have been proposed as an instrument for clinical diagnosis in disciplines such as radiology, histology, ophthalmology, and dermatology (2).

The rapid spread of CNNs and other deep learning techniques, has created the need for non-experts to become familiar with these complex tools and understand their principles of operation. There is a broad literature of introductory articles offering basic reviews on CNNs principles and applications. However, on the practical side, tutorials to start working with CNNs often require a familiarity with terms and concepts that could discourage readers without a solid background in mathematics and/or computer programming to obtain a further understanding of these techniques.

In this scenario, we aim to close this gap by offering a hands-on activity based on the step-by-step execution of a computer code involving all the procedures carried out when implementing a CNN classification, along with their description. Similar educational activities have been recently proposed for other research fields (3). The activity guides the student through two complete examples based on images of skin lesions, starting from the pre-processing of the dataset, and leading through the steps of data augmentation, the choice of the network architecture and its fine-tuning, until the final evaluation of the results.

In our opinion, this hands-on activity can help students to obtain an intuitive understanding of the operations performed by the CNN building blocks, e.g., by visualizing the effect of image convolution with a specific kernel, the feature map generated at specific network layers, or even by performing the network training and exploring the effect of different hyperparameters on the results. The activity can be performed at different levels of difficulty, depending on the user expertise in programming. At the basic level of execution, the students can interactively play with the different sections just by changing input parameters from simple form fields and can run the program by pushing the play button, without even visualizing the code. At the intermediate/advanced levels, students can unfold cells to show, read and (possibly) modify portions of the code. In both cases, the use of the Google Colab and GitHub platforms allows to run the activity in the cloud from any internet browser, without any software installation, strongly simplifying configuration requirements and enabling the capability to use hardware accelerators.

## Pedagogical Framework and Learning Environment

We aimed at developing a hands-on activity mainly directed to students (medical school, biomedical engineering), but with the potential of being of interest also for clinicians and other professionals willing to get acquainted with deep learning and CNNs. Four major developments make such a learning-by-doing experience nowadays possible, even for non-experts. First, the creation of specific software libraries, which have reduced the complexity and length of the code necessary to implement these networks, thus allowing their use to operators with a basic knowledge of computer programming. Secondly, the distribution of pre-trained classical CNN under license for reuse has enabled the possibility to perform transfer learning further simplifying the coding and speeding up the training. Third, the free availability of cloud computing on virtual machines with graphics (GPUs) and tensor processing units (TPUs), which has played an important role in speeding up training procedures. The last development of note is the accessibility of databases containing labeled images for training.

Along these lines, for the proposed activity we use Keras (4), an open-source framework developed by Francois Chollet. Several open-source frameworks are nowadays available for deep learning such as PyTorch or Caffe. However, Keras is recommended for beginners since it is relatively easy to use and has a high-level API that permits to build complex models by writing a few lines of code. In addition, Keras has several models of the best performing architecture (Alexnet, ResNet, VGGNet, Inception, etc.) pre-trained on large datasets (e.g., the one used for the ImageNet Large Scale Visual Recognition Challenge (ILSVRC, http://image-net.org) containing millions of photographs from 1,000 categories) that have thus achieved very general classification capabilities. These trained networks can be “reconverted” to the classification of different target images in few simple steps and their learnable parameters (weights and biases) are fine-tuned to provide high classification accuracy, through a procedure called transfer learning.

The code for the activity is provided on the Google Colaboratory platform (Colab, https://colab.research.google.com/notebooks/intro.ipynb). Colab is a free cloud service that enable coding in Python and program execution in a web browser, in a highly interactive fashion. In addition, it requires a minimum number of configuration steps, offers free access to GPUs and TPUs, and allows sharing of contents in a straightforward manner. The notebooks and metadata necessary for the activity are shared on GitHub (www.github.com), a free hosting service for software development and version control.

Deep learning requires a massive amount of information in the form of labeled images. Several repositories contain high-quality images associated to dermatology, available as research tools in clinical training and computer science. For example, the archive of the International Skin Imaging Collaboration (ISIC, https://isic-archive.com/), or the Edinburgh Dermofit Library (https://licensing.edinburgh-innovations.ed.ac.uk/i/software/dermofit-image-library.html) host images of skin lesions, labeled according to their diagnoses. For the activity, we use images from the dataset that has been recently made available for the training of methods competing for the ISIC 2019 challenge (https://challenge2019.isic-archive.com), a competition aimed at supporting research toward automated melanoma detection. The full dataset contains 25,331 images of skin lesions associated to 8 different diagnostic categories (melanoma, melanocytic nevus, basal cell carcinoma, actinic keratosis, benign keratosis, dermatofibroma, vascular lesion, and squamous cell carcinoma) and can be accessed by registering to the ISIC website.

## Description of The Hands-on Activity

The files to perform the activity are stored on the GitHub public repository https://github.com/qubilab/CNN-for-skin-lesion-classification. The two links, associated to examples of a binary (benign/malignant) and a multiclass (melanoma/melanocytic nevus/basal cell carcinoma/actinic keratosis/benign keratosis/dermatofibroma/vascular lesion/squamous cell carcinoma) classification of images of skin lesions, automatically redirect to the respective Colab notebooks.

For the basic use of the notebook, no configuration is needed. For applications requiring hardware accelerators (e.g., training or fine-tuning), GPU or TPU can be enabled by picking the required accelerator from the menu that appears by selecting Runtime/Change runtime type.

For a better pedagogical support, the notebook is organized in consecutive sections, each with a brief explanation of the task performed, that guide the user steps-by-step along the activity. To execute the code in a cell, it is sufficient to select it with a click and then either press the play button within the cell box or use the keyboard shortcut “Command/Ctrl+Enter.” For more advanced applications, the visualization of input form fields or code is achieved by unfolding the cell content, by clicking on the little arrowhead at the left of the cell, thus enabling the necessary editing.

### Image Pre-processing and CNN Basics

**Figure 1:**
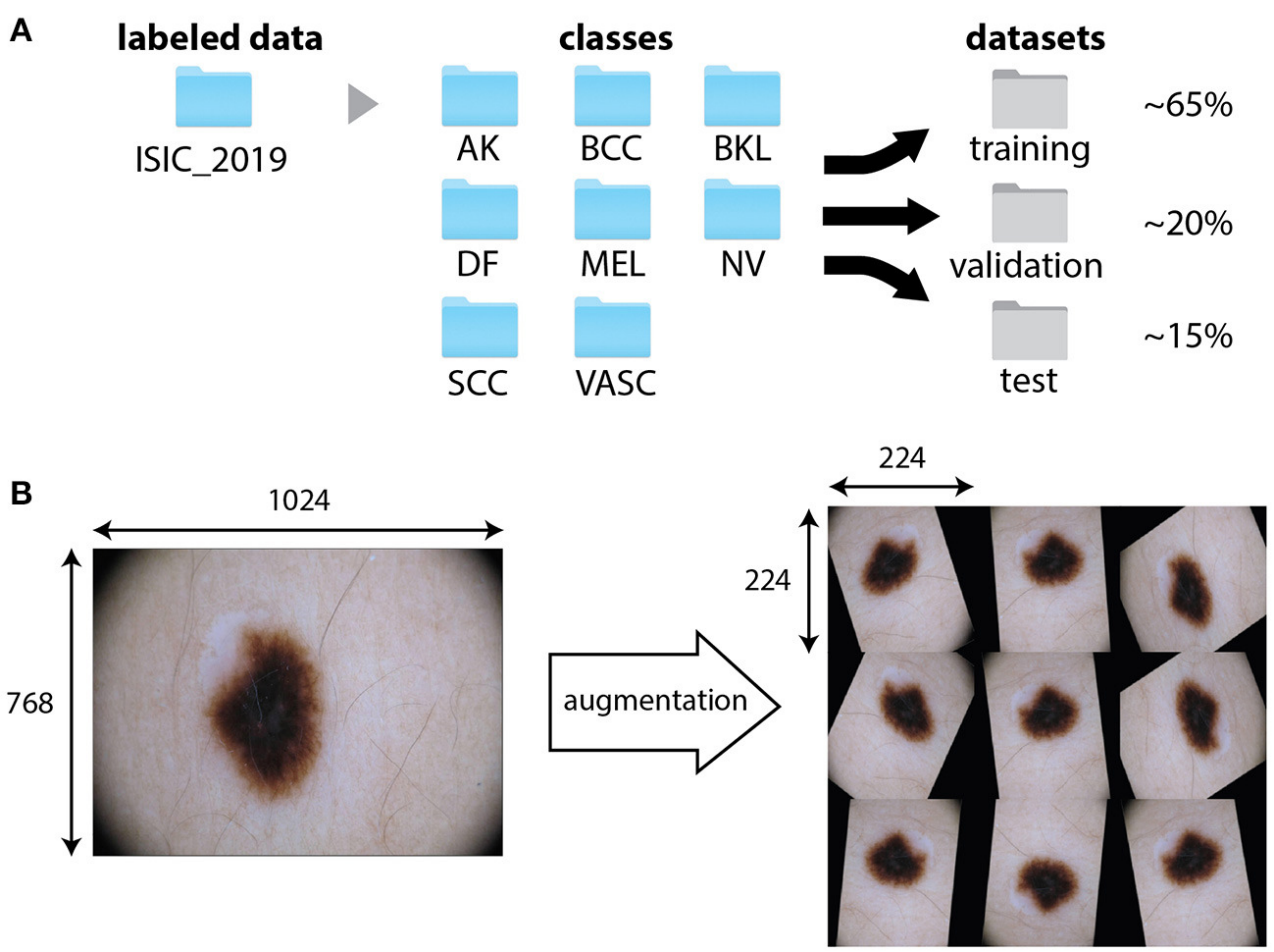
Data pre-processing and augmentation. **(A)** Example of the organization of the training dataset ISIC 2019. Images are first divided into folders associated to the different categories. A specific function split them randomly into training, validation and test datasets. **(B)** An original image from the training dataset ISIC 2019 (class NV) and possible outputs of the application of the augmentation procedure, consisting in combinations of random shift, rotation, and reflection. The data augmentation function resizes the images to fit the input size of the network (224 × 224 × 3).

The first cell “0. Imports” gives access to the code provided in other modules and libraries and defines some basic functions. Once this operation has been performed, we enter into the core of the activity. In fact, since most of the deep learning approaches are data-driven, a major focus must be set on the dataset and its organization. The cell “1. Loading and organizing the dataset” loads the labeled images on the Colab cloud space and arrange them into folders (Figure 1A). The first block of this section requires the input of user credential to login on the ISIC archive and the selection of the number of images per category. The photographs will then be organized into folders according to their category and further randomly split into training, validation, and test sets. A variable percentage of images (10–30%) can be used for the final testing, whereas the others are split between training (70–80%) and validation. The folder tree can be visualized in the left frame of Colab, by selecting “Files.” In the example, we use percentages of 65, 20, and 15% of the total number of images for training, validation and test, respectively. These percentages can be changed by the user through the form fields of block 1.3.

The cell “2. Understanding images and convolution” allows the user to visualize an image from a selected folder, together with its decomposition into layers according to RGB color model and the representation of the pixel intensity value. To provide an intuitive understanding of the principle of CNN, the activity shows different convolution kernels and their pixel values. The convolution of the image with these kernels is further provided. An introductory description of the convolution and of the hyperparameters of a convolutional layer can be found in our previous article (5).

Often, the limited amount of data available for training might produce the memorization of specific details of the training images, leading to overfitting and the inability of the model to generalize. In this case, it is recommendable to perform a procedure called data augmentation. The augmentation generates modified images by applying random transformations, such as rotation, shift, scaling, and reflection, to existing data (Figure 1B). Typically, the computer function used for augmentation also takes care of resizing the images to the input size required by the network. These steps can be visualized on a random image by executing the cell “3. Data augmentation.”

### CNN Selection

Once the data have been obtained and properly organized, the following step entails the choice of the classification network. In principle, users could build their own network by assembling it layer-by-layer. Application program interfaces allow one to create a CNN from scratch relatively easily. However, this is generally not recommended for beginners, since it requires some background knowledge, a good dose of intuition, and some trial and error. Moreover, unless one is facing a new and very specific image classification task, a personalized CNN is often not needed: many popular deep learning architectures are released under a permissive license for reuse. Even in the case that it is essential to build a custom model, classic networks might still serve as an inspiration, a scaffold, or as a block of the new model. Nowadays, several networks offering outstanding performance for image classification are available, therefore choosing the most suitable CNN for one's application is not straightforward. Since these CNNs have been originally built for applications on different datasets, the selection should be based on their performance on the target dataset and thus requires their evaluation and comparison (6).

### ResNet-50

We use the CNN ResNet-50 (7). ResNet architectures were developed by the Microsoft Research team (7) and are available in several versions with different number of layers, such as 50, 101, 152 (https://github.com/KaimingHe/deep-residual-networks). Notably, the ResNet-152 won the 1st places in all the sections of the ILSVRC and COCO (http://cocodataset.org/#detection-2015) competitions in 2015. Pre-trained ResNet architectures have been frequently used for the classification of skin lesions, even by several participants to ISIC challenges (8–12).

**Figure 2:**
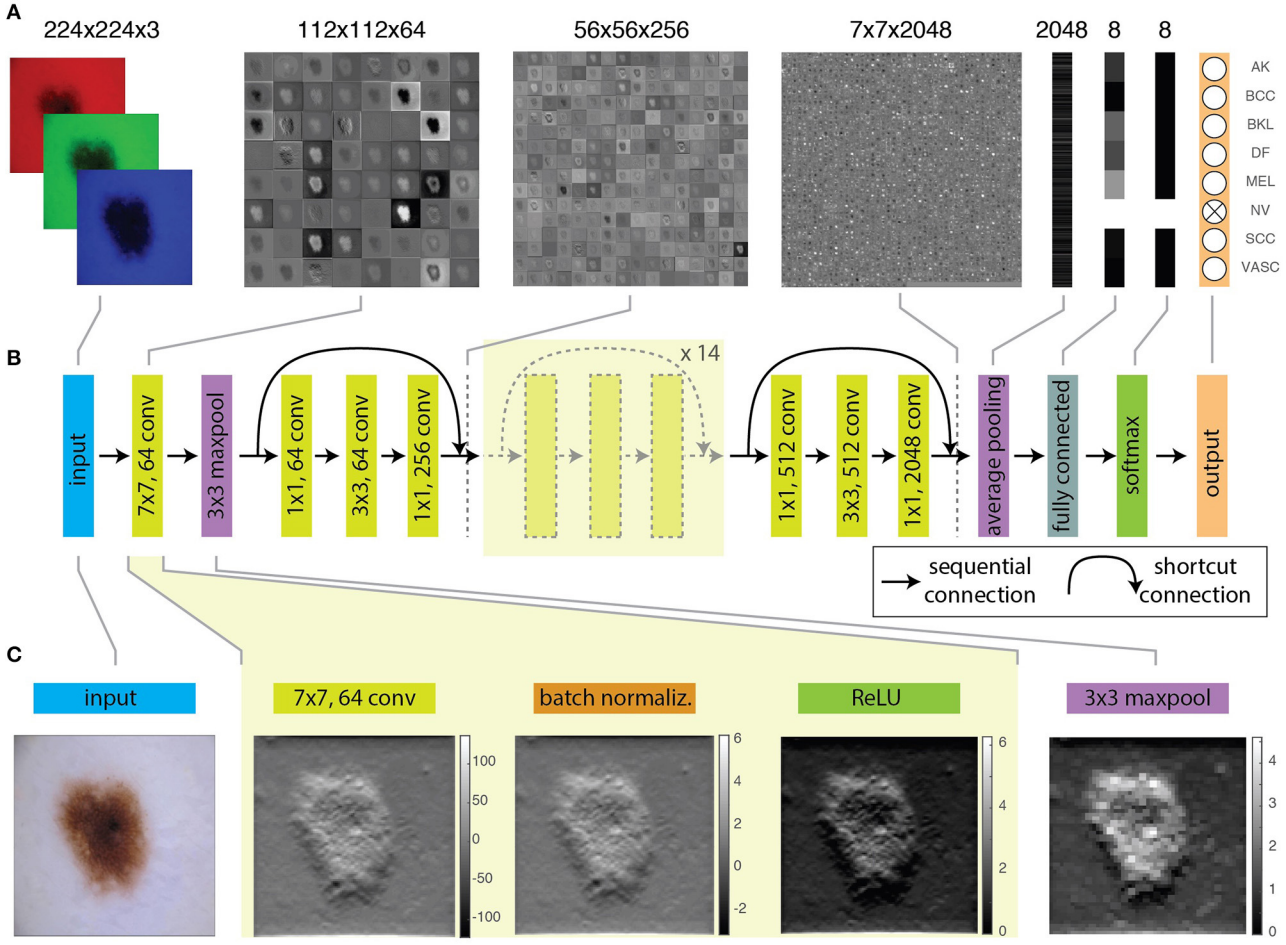
Schematic and operations of a ResNet-50. **(A)** Feature maps obtained at different layers of a trained ResNet-50, from the input RGB image, to the 8-element probability vector predicting the image class. **(B)** Schematic illustration of representative layers of the network. **(C)** Effect of convolution, batch normalization, activation, and maxpooling on 1 of the 64 features produced by the first convolutional layer from the input image.

A schematic representation of the architecture and functions of ResNet-50 is shown in Figure 2. Figure 2A shows examples of feature maps obtained at specific layers. Moreover, the code of the hands-on activity displays the feature map for any selected layer of the CNN (cell “8.5 Visualize features generated at a specific layer”). Figure 2B contains a scheme of the layers and the connections of the network. Figure 2C shows the effect of the application of specific operations (convolution, batch normalization, activation and maxpooling) on an image.

As shown in Figure 2A and in cell “2. Understanding images and convolution,” an image is a collection of two-dimensional matrices (channels) which elements (pixels) have numeric values representing the brightness in each channel. Color images are composed of multiple channels (e.g., 3 for the RGB representation) whereas grayscale images only have one. The application of the network over an input image, produces its progressive transformation into a larger number of features with smaller lateral dimensions. Eventually, the features are combined to obtain a set of scalar values with the same dimension of the output categories. This is achieved through the repetition of mathematical operations performed by the layers and depending on a large number of learnable parameters.

The input is first processed by a convolutional layer. Convolution is a mathematical operation involving the input image and a kernel, typically a matrix with smaller lateral dimensions with respect to the input. During CNN training, kernels are randomly generated and each kernel produces a different feature. Convolution is performed by sequentially shifting the kernel along the input image by a fixed number of pixels, called stride. At each step, the sum of the element-by-element multiplication between overlapping pixels returns the pixel value of the convolutional feature. In cell “2. Understanding images and convolution” the output provided by different kernels can be interactively explored. It must be pointed out that a stride larger than one can be used to obtain features with reduced lateral size with respect to the input.

The first convolutional layer of the ResNet-50 uses 64 kernels of 7 × 7 pixels^2^ with a stride of 2 pixels and thus produces 64 features with half the lateral size of the input image (Figure 2). The features are regularized through a batch normalization layer (Figure 2C), that performs the standardization of the input, corresponding to the subtraction of the mean of the batch and the division by the standard deviation. The convolutional and batch normalization layers are generally followed by an activation function that performs a non-linear transformation of the feature map, providing the input for the next convolutional layer. Non-linear functions (like sigmoid or hyperbolic tangent) are used as activation functions for their similarity to the behavior of real neurons, i.e., the transformation of a continuous input into a digital output. The most widely used function is the rectified linear unit (ReLU). The ReLU performs a simple calculation: it returns the same value provided by the input if the input is positive. However, if the input value is negative or null, it returns zero. Inputs that are converted to zero constitute non-activated neurons. In this way, not all neurons are firing simultaneously. The sparse activation and the simpler mathematical operation guarantee a higher computational efficiency for ReLU as compared with other non-linear functions.

The batch normalization and the activation layers preserve the lateral size of features obtained at the output of the convolution layer. However, it is often recommendable to create a lower resolution version (downsampling) of the output image to reduce the number of parameters and account for variations in the position of features in the input image. A common approach is to use a pooling layer, which substitutes adjacent subregions of specific size with the sum, average or maximum values of the corresponding pixels. The ResNet-50 uses a maxpooling layer (Figure 2C) to downsample images by taking the maximum of the input over 3 × 3 regions.

Combinations of these layers are applied along the network, progressively reducing the lateral size from 224 × 224 to 7 × 7 and increasing the depth of the feature map from 3 (the RGB layers of the input image) to 2048. At this point, a group of three layers flattens the feature map, i.e., transforms it into a score vector with the same length as the number of categories. The values of elements of this vector correspond to the probability that the input image belongs to each category (Figure 2B) and the maximum of this vector will correspond to the category assigned to the input image by the CNN. The first layer of the group performs a downsampling through average pooling, a pooling operation returning the mean of the region of the image considered. By using an average pooling over 7 × 7 regions, each of the 2048 features is thus reduced to a single value given by the average of all the pixels in the 7 × 7 map (Figure 2A).

The further reduction of the number of elements of the vector to a size equal to the number of categories is obtained through the fully connected layer. Each output value of this layer has a complete connection with all the 2048 inputs, as it is obtained as their weighted sum. The very last layer normalizes these values into the probabilities to belong to each labeled category. Usually, this task is performed through a softmax function that generalizes binary logistic regression to the case of a multiclass problem.

### Training

Once the dataset is ready and the CNN has been chosen, it is possible to start the actual training of the network. During this procedure, values of the learnable parameters are randomly changed, and the corresponding features are calculated to provide a tentative classification of the images in the training set. The performance of the network is evaluated by the calculation of a metric (loss function) that quantifies the similarity between the prediction and the ground-truth. Parameters are iteratively adjusted to optimize the loss function and thus increase correct predictions.

However, as mentioned earlier, we need to distinguish between two different situations. The first refers to the case in which the network needs to be fully trained. In this case, the values of all the parameters of the network need to be learned from scratch. This procedure requires very large datasets, often not available for medical applications. However, the use of a classical network further enables the possibility of performing transfer learning: besides using the same architecture as a classical network, one can also take advantage of parameters learned by the previous training of the CNN on a different, larger dataset. In transfer learning, the parameters obtained from the training of the model over a large dataset are only fine-tuned to adapt the network for the classification of a different target dataset. In this way, one can skip the time-consuming training steps but still take advantage of the features learned from the training over many photographs. As reported in (6), the top-performing methods submitted for ISIC challenges 2016, 2017, and 2018 used CNNs pre-trained on the ImageNet database. In cells 4–6, we perform these steps using the ResNet-50 pre-trained on the ImageNet dataset.

The ResNet-50 is designed for the ImageNet challenge and thus its output is composed of 1,000 categories. To use the ResNet-50 to classify skin lesion images among a different number of classes, it is first necessary to replace the last layers by ones providing an output over the desired number (2 or 8) of categories (Figure 2B). This procedure is performed in the section “4. The CNN: ResNet-50.”

### Hyperparameters

The CNN will learn weights and biases by minimizing the loss function over the training set using a method called stochastic gradient descent. However, due to the large size of the dataset and the limited memory, it is not possible to feed all the images simultaneously to the CNN. Therefore, training images are generally passed to the CNN in smaller groups called batches (cell 4.1). The optimum batch size must be set by trial and error in order to provide the fastest convergence. As a rule of thumb, small but not too small batch sizes (e.g., 32, 64, 128) are preferred, since they show higher accuracy than very large batches (13). The number of iterations necessary for the network to “see” the entire training dataset constitutes an epoch.

Another relevant hyperparameter is the global learning rate (cell 5.1), a number between 0 and 1 determining the step size used to update the weights at each iteration. The learning rate sets the speed at which the model is adjusted to the data. A low learning rate applies small changes to the weights at each update, thus requires more epochs of training. Although a high learning rate produces faster changes, if too high it might not converge to an optimal model. The correct learning rate should be empirically chosen to obtain convergence in a reasonable amount of time. Typically, the learning rate is not fixed but is progressively reduced during the optimization. Large rates are first used to quickly obtain values of the weights corresponding to a loss function close to its minimum. At that point, smaller rates further adjust the weights to better approximate the exact minimum of the loss function (cell 5.2). In addition, since in CNN the features provided by the early layers are more generic, whereas those belonging to the last layers are dataset specific, one can introduce non-uniform learning rates and either “freeze” the early layers or train the new layers at a faster rate with respect to the others (cell 5.1 and 5.3). Besides, several other hyperparameters need to be set in relation to the optimization procedure. In the activity, we adopt a procedure and use hyperparameters similar as those described in (11).

### Optimization

The algorithm is now ready to start the optimization process, a procedure involving the minimization of a loss function that measure the distance between the predicted and the ground-truth classification. For classification tasks, the cross-entropy function is the usual choice (cell 5.4). The optimization will run until some convergence condition is met. This condition is set by the user based either on the value of accuracy/error calculated on the validation set, or on a maximum number of validations without improving the loss value (cell 5.2).

**Figure 3:**
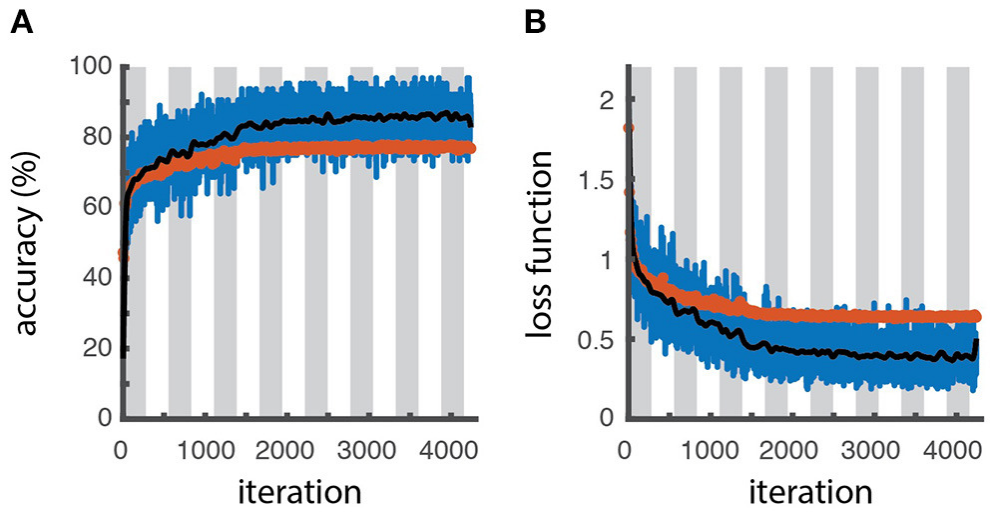
Learning curves. Accuracy **(A)** and loss function **(B)** as a function of the number of iterations for the training batch (blue line for raw data, black line for filtered data) and validation sets (red symbols), as obtained through the optimization procedure. The white and gray areas delimit different epochs. The slightly higher accuracy and lower loss values obtained for the training dataset respect to the validation one reveals a slight overfitting.

The actual training is performed in section “6. Fine-tuning the model.” The learning process can be visually monitored by displaying the trend of learning curves calculated from both the training and validation datasets as a function of algorithm iterations, to have an idea about how well the model is, respectively, learning and generalizing (defined in cell 5.5). Typically, plots of learning curves associated to optimization (e.g., cross-entropy loss) and performance (e.g., accuracy) are simultaneously created for both datasets (Figures 3A,B). The comparison of learning curves obtained for training and validation datasets is a valuable diagnostic method for the model behavior. A training loss showing a continuous decrease with a validation loss showing a minimum, in general correspond to an overfitting model. A good fit is usually associated with training and validation losses decreasing simultaneously toward closely spaced horizontal asymptotes (4). However, a higher accuracy (lower loss) of the training set with respect to the validation can still indicate some degree of overfitting, as obtained for the example shown in Figures 3A,B.

Since the training can sometimes run for quite a long time, in section “7. Load a trained network” we also provide a previously-trained model with weights. By loading this model, the user can perform the remaining part of the activity without having to wait for the completion of the training procedure.

### Performance Evaluation

**Figure 4:**
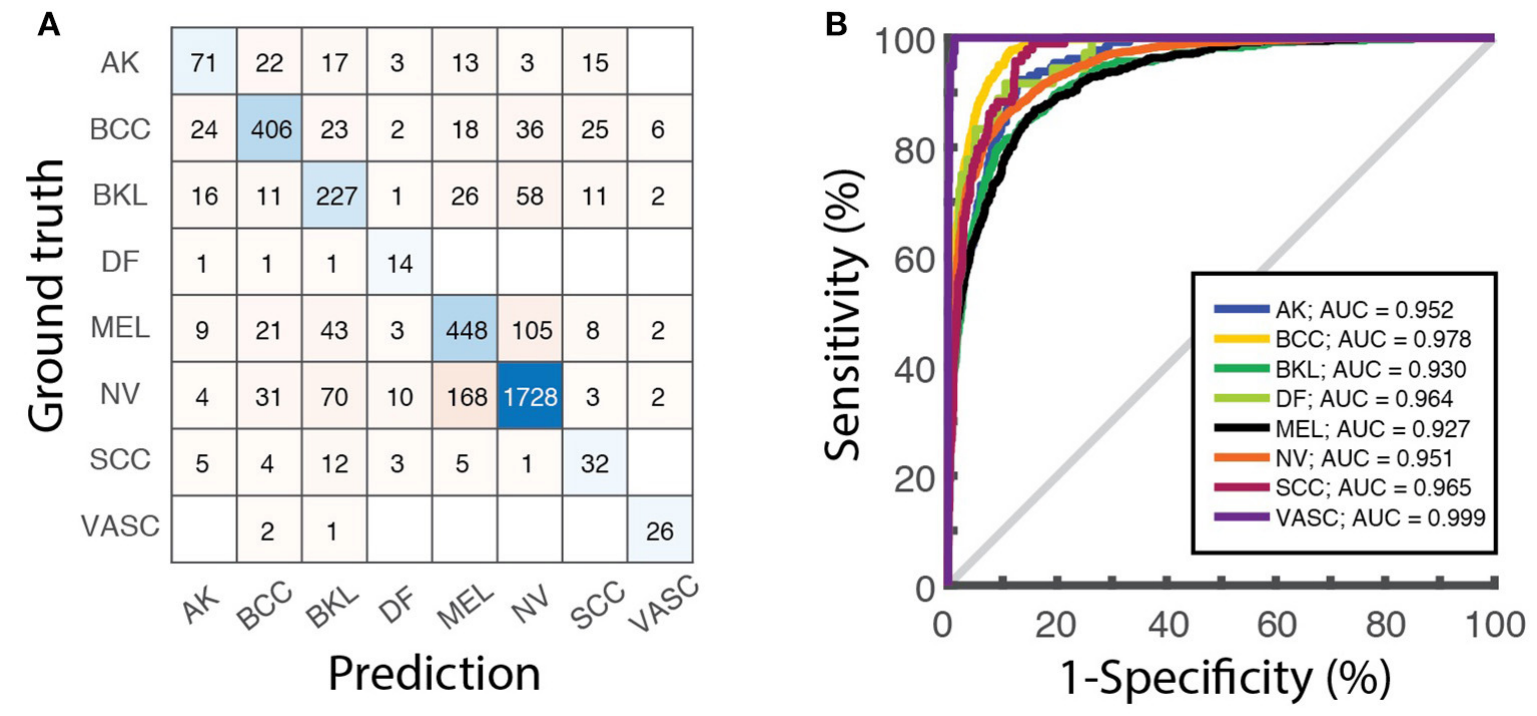
Model evaluation over the test dataset. **(A)** Confusion matrix displaying the number of images of the test dataset associated to each category by the ResNet-50 network. **(B)** ROC curves obtained on the same dataset and the corresponding values of the AUC for each class.

Once the learning phase is complete, the network can be finally applied to predict the class of the images contained in the test dataset, that were not used for the training. Since the ground-truth of these images is known, they can be used to calculate metrics to assess the performance of the classifier (section “8. Performance assessment”). Besides the overall accuracy, the confusion matrix, reporting the number of correct and incorrect predictions over all the classes, (Figure 4A) visually summarize classification and mis-classification performance of the model. The evaluation of the model performance further includes the plot of the receiver operating characteristic (ROC) curves and the calculation of the area under the curve (AUC) for each category (Figure 4B), obtained as detailed in (5).

## Discussion and Conclusions

We have developed a hands-on activity based on the interactive computer code and a detailed description of the steps needed to implement and fine-tune a CNN to perform the classification of dermatological images, together with an intuitive explanation, suitable for non-experts, of the functions performed by the main blocks of the network. The description is based on two practical examples, consisting of the fine-tuning of a pre-trained ResNet-50 network on a public dataset, containing images of skin lesions corresponding to different diagnoses. The use of *ad hoc* toolboxes and libraries largely simplifies the coding and makes it accessible to beginners.

In our opinion, the hands-on example, together with the description provided in this article, can act as a tool for students interested in obtaining a first understanding of the inner working of a CNN. However, the same activity can also be offered to provide a tutorial for beginners' initiation to computer programming for building and optimizing CNNs. In the first case, the code can be simply executed with the default parameters to visualize the output of each cell. The visualization of the results provides an intuitive understanding of CNN principles. As an example, plotting the feature maps obtained at consecutive layers allows comparing the changes introduced on the features by pooling, batch normalization and activation layers. In the second case, the user can further explore how modifications of the dataset and the change of hyperparameters affect the network's performance. Examples in this sense might involve the comparison of performance upon the change of learning rates (or even the freezing) of specific layers.

The use of an interactive hands-on activity reproducing a novel approach in its complexity might be a powerful strategy to approach the development of problem-solving and analytical skills, possibly through group work in the classroom. In addition, we believe that making this technology more accessible for non-expert will contribute to further strengthen the collaboration between dermatologists and computer scientists, toward the joint effort of improving image-based medical diagnosis.

## Author Contributions

CM and MC-D contributed to conception and design of the study. SN, IM, and CM wrote the code and performed analyses. MC-D wrote the first draft of the manuscript. CM wrote the final version of the manuscript. CM and MO-V supervised the research. All authors contributed to manuscript revision, read, and approved the submitted version.

## Conflict of Interest

The authors declare that the research was conducted in the absence of any commercial or financial relationships that could be construed as a potential conflict of interest.
